# A horizontal gene transfer supported the evolution of an early metazoan biomineralization strategy

**DOI:** 10.1186/1471-2148-11-238

**Published:** 2011-08-12

**Authors:** Daniel J Jackson, Luciana Macis, Joachim Reitner, Gert Wörheide

**Affiliations:** 1 Courant Research Centre Geobiology, Georg-August-University of Göttingen, Goldschmidtstr. 3, 37077 Göttingen, Germany; 2 Department of Earth- and Environmental Sciences & GeoBioCenterLMU, Ludwig-Maximilians-Universität München, Richard-Wagner-Str. 10, 80333 München, Germany; 3 Bavarian State Collections of Palaeontology & Geology, Richard-Wagner-Str. 10, 80333 München, Germany

## Abstract

**Background:**

The synchronous and widespread adoption of the ability to biomineralize was a defining event for metazoan evolution during the late Precambrian/early Cambrian 545 million years ago. However our understanding on the molecular level of how animals first evolved this capacity is poor. Because sponges are the earliest branching phylum of biomineralizing metazoans, we have been studying how biocalcification occurs in the coralline demosponge *Astrosclera willeyana*.

**Results:**

We have isolated and characterized a novel protein directly from the calcified spherulites of *A*. willeyana. Using three independent lines of evidence (genomic architecture of the gene in *A. willeyana*, spatial expression of the gene product in *A. willeyana *and genomic architecture of the gene in the related demosponge *Amphimedon queenslandica*), we show that the gene that encodes this protein was horizontally acquired from a bacterium, and is now highly and exclusively expressed in spherulite forming cells.

**Conclusions:**

Our findings highlight the ancient and close association that exists between sponges and bacteria, and provide support for the notion that horizontal gene transfer may have been an important mechanism that supported the evolution of this early metazoan biomineralisation strategy.

## Background

**Figure 1:**
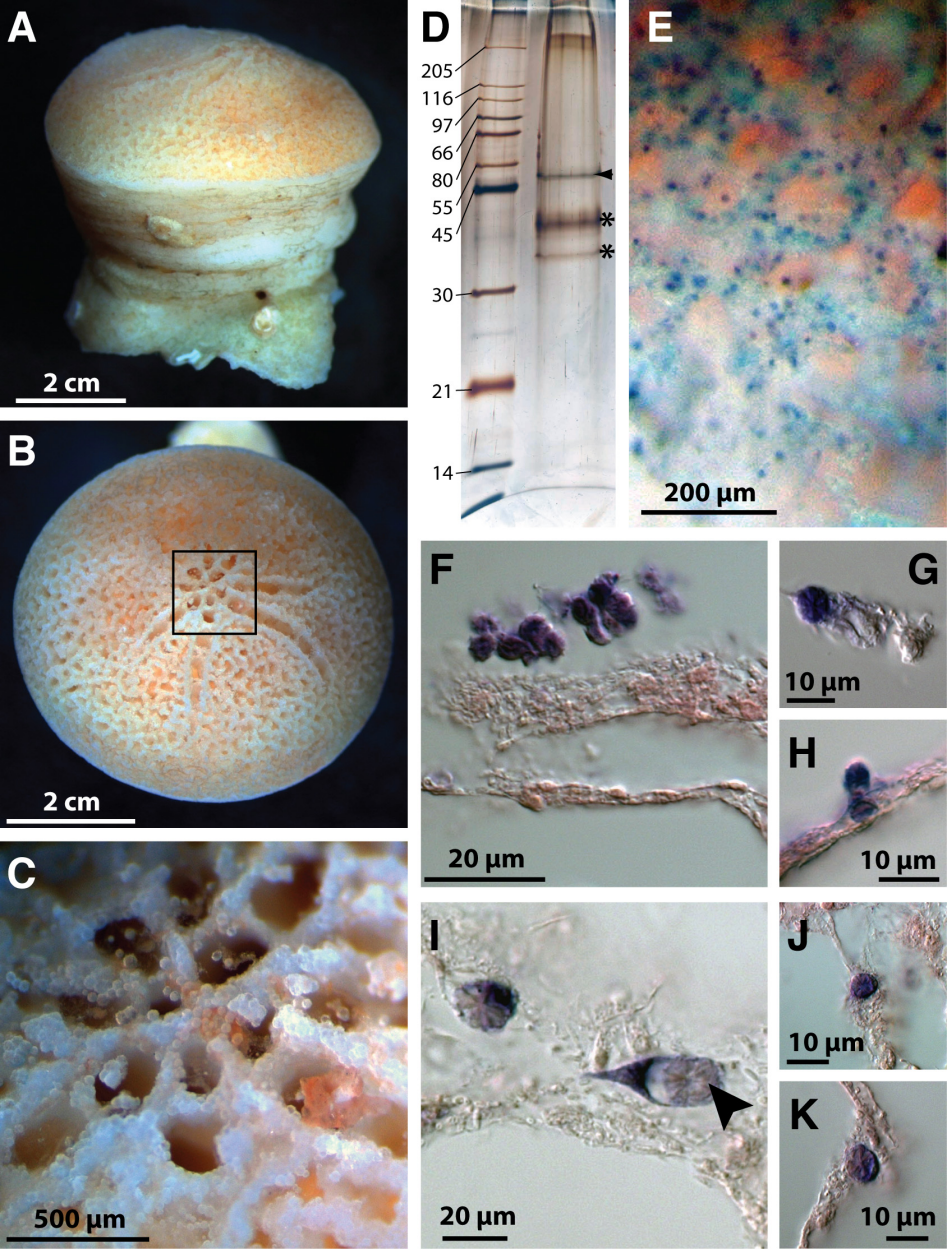
***A. willeyana *and characterization of *Awi-Spherulin *expression**. (*A*) Lateral and (*B*), apical views of *A. willeyana*. (*C*) Expanded view of the boxed region in (*B*) illustrates nascent spherulites. (*D*), SDS-PAGE gel of organic material extracted from purified spherulites. The band at 52 kDa (arrow) is *Awi*-Spherulin. Bands indicated by asterisks are Astrosclerin. (*E*) Apical view similar to that in (*C*) following WMISH against *Awi-Spherulin*. Positive cells have a distribution indicative of a role in spherulite formation. (*F*-*H*) Sections of WMISH preparations show *Awi-Spherulin *is expressed within spherulite forming cells. (*I-K*) WMISH against *Astrosclerin *reveals the same expression pattern as *Awi-Spherulin*. Arrowhead in (I) indicates the insoluble organic matrix of an individual spherulite.

The fossil record of early animal life could be briefly summarized as a period dominated by soft-bodied organisms that were replaced at the dawn of the Phanerozoic by an unprecedented diversity and disparity of animals able to form mineralized structures [1-4]. Decoding the genetic and molecular mechanisms that instruct the assembly of mineralized body parts is therefore of great importance if we are to have a complete understanding of how complex animal life evolved. Because sponges evolved the capacity to construct a skeleton early on in their evolution [1], and are widely thought to be the earliest branching metazoan phylum [5,6], we have been investigating the genetic and molecular basis of biocalcification in the coralline demosponge *Astrosclera willeyana *[7,8]. Some coralline sponges, such as *A. willeyana*, construct their calcified skeletons by accreting individual microscopic spherulites together (Figure 1a-c), a strategy employed by reef forming sponges known from the Permian and Triassic [9]. Recently we demonstrated that spherulites in *A. willeyana *are initially formed by calcifying the organic remains of degraded bacteria [8] via the activity of at least one highly conserved biomineralizing enzyme [7]. Here we show that a major proteinaceous component of the *A. willeyana *skeleton, which is likely to be directly involved in spherulite formation, was horizontally transferred from a bacterium into the *A. willeyana *genome.

## Results and Discussion

### Isolation and evolutionary characterization of the novel biocalcification protein Spherulin

Using previously described de-mineralization, protein extraction and separation techniques [7], our continuing efforts to characterize the spherulitic proteome of *A. willeyana *identified a prominent protein of 52 kDa on SDS-PAGE gels. This band was excised, and an amino terminal fragment sequenced by Edman degradation. The resulting peptide was used to design degenerate PCR primers which amplified a fragment of DNA from a cDNA library. This was cloned, sequenced and used to design gene specific primers that allowed the full-length cDNA to be isolated (Additional file 1). Due to the association of this protein with spherulites we have named it 'Spherulin'.

Initial BLAST searches using partial RACE fragments returned hits against uncharacterized proteins with similarity to sugar transporters derived exclusively from bacteria. Conversely, exhaustive BLAST searches directed against a wide phylogenetic range of eukaryotic unicellular and multicellular draft or complete genomes (more than 270) did not return any positive hits (see Additional file 2 for a complete list of eukaryotic genomes searched). One exception to this was the identification of a clear Spherulin homolog in the draft genome of the demosponge *Amphimedon queenslandica*. This restricted phylogenetic distribution of Spherulin led us to the initial hypothesis that the poriferan Spherulin sequences were in fact derived from the abundant bacterial communities known to exist throughout *A. willeyana *and *A. queenslandica*, and which are particularly numerous within the cells that initiate spherulite formation in *A. willeyana *[8,9]. However, once we isolated the full length mRNA from *A. willeyana *the presence of a putative poly-A signal and poly-A tail (Additional file 1) suggested that this may not be a bacterially derived gene product, and could be the product of a horizontal gene transfer (HGT).

### Three independent lines of evidence demonstrate *Spherulin *is now located within poriferan genomes

To test this hypothesis we first localized *Awi-Spherulin *expression using whole mount *in situ *hybridization (WMISH). These experiments clearly show that *Awi-Spherulin *is expressed in the same spherulite forming cells that *Astrosclerin *is expressed in (Figure 1f-k), a gene we previously demonstrated to be involved in spherulite formation [7]. Furthermore, endobiotic bacteria associated with *A. willeyana *are visible following WMISH, and *Spherulin *expression was not detected in these bacterial cells after multiple rounds of WMISH (data not shown).

**Figure 2:**
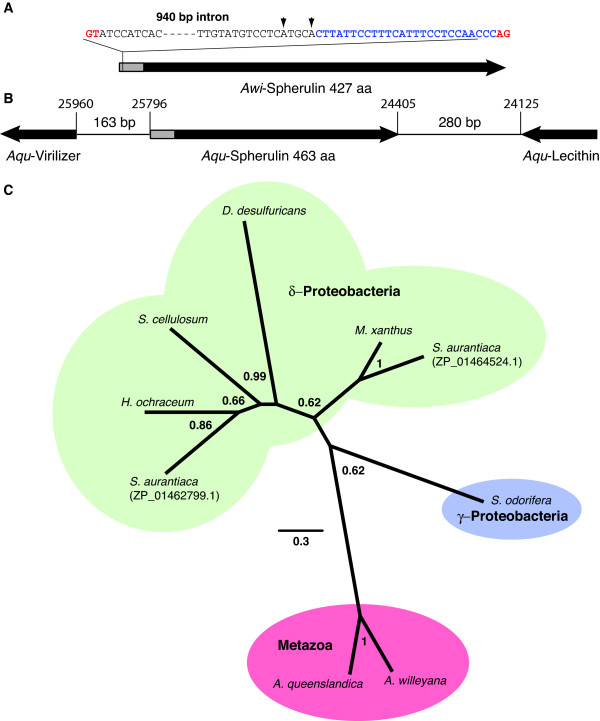
**Genomic architectures of *Awi- *and *Aqu-Spherulin *and their evolutionary relationships with homologous prokaryotic sequences**. (*A*) *Awi-*Spherulin possesses a signal sequence (highlighted grey) indicating it is located extracellularly, within which a 940 bp intron is located. GT/AG splice sites (highlighted red), a polypyrimidine tract (highlighted blue) and putative adenine break point residues (arrows) are indicated. (*B*) *Aqu-Spherulin *is located on genome scaffold 13507 (schematically represented here reverse complimented), and is immediately flanked by eukaryotic specific sequences. Scaffold locations are indicated. (*C*) Bayesian phylogenetic analysis of eukaryotic and prokaryotic Spherulin sequences.

We also investigated the genomic architectures of *Awi- *and *Aqu-Spherulin*. We amplified *Awi-Spherulin *from genomic DNA and found that the amplicon was 940 bp larger than the cDNA derived product. The 940 bp of additional sequence possessed the characteristics of a typical spliceosomal intron with classic GT-AG donor/acceptor sites [10], a polypyrimidine tract 5' of the acceptor site [11], and several adenine residues immediately upstream of this tract that could act as intron break point residues [12] (Figure 2a). The presence of this splicesomal intron indicates that the *Awi-Spherulin *gene is now located within the *A. willeyana *genome.

While the *A. queenslandica Spherulin *homolog is intron-less, genomic sequence immediately flanking *Aqu-Spherulin *codes for metazoan specific genes (*Virilizer *and a lecithin:cholesterol acyltransferase), indicating that *Aqu-Spherulin *is located within the *A. queenslandica *genome (Figure 2b).

### The deep ancestry of the *Spherulin *HGT event has removed evidence of *Spherulin's *bacterial heritage

Phylogenetic incongruence, where evolutionary relationships are well established, can indicate a history of HGT activity [13], and is considered to be a gold standard for the demonstration of HGT ancestry [14]. We therefore performed phylogenetic analyses using a range of Spherulin sequences. All of our analyses grouped the two poriferan Spherulins together with high support, while all other prokaryotic sequences formed distantly related clades (Figure 2c). While this result is not phylogenetically incongruent (sponge Spherulin proteins would be expected to form an outgroup to all other prokaryotic Spherulin-like sequences in the absence of HGT), it does not provide evidence against HGT. It does however, provide information regarding the origins of the poriferan *Spherulin *gene. Because *Awi- *and *Aqu-*Spherulin form a well separated and supported clade, it is clear that an HGT event established this gene in a common ancestor of *A. willeyana *and *A. queenslandica*, and must therefore have occurred during or prior to the Triassic 265-220 MYA when these two species of sponge shared a common ancestor [9]. Because of the long branch that leads to the two poriferan genes, they must have since been evolving under pressures distinct from those of the prokaryotic Spherulin homologs.

**Figure 3:**
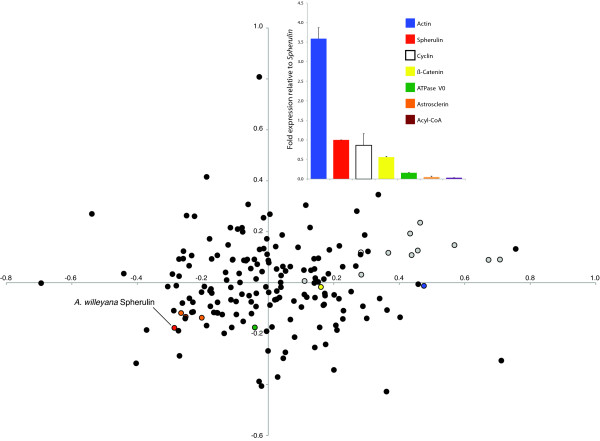
**Comparative qPCR analysis and codon usage bias of *A. willeyana *and prokaryotic genes**. The bar chart illustrates the expression levels of six *A. willeyana *house keeping genes quantified relative to *Awi-Spherulin*. Error bars are the standard error of 3 replicates derived from 3 individuals. The scatter plot is a correspondence analysis of relative synonymous codon usage (RSCU) of 181 *A. willeyana *genes. Superimposed are the RSCUs of 10 prokaryotic *Spherulin *homologs (grey circles) and *Awi-Spherulin *(red circle). Also indicated by color are the genes used in the qPCR analysis.

Further evidence for the deep ancestry of the *Spherulin *HGT is provided by a composition analysis. Codon usage typically differs significantly between prokaryotic and eukaryotic organisms [15], and can therefore be used to detect cases of HGT [16-18]. The level of gene expression often influences codon usage bias, with highly expressed genes typically possessing a greater bias [19]. Accordingly, we first quantified levels of *Spherulin *expression from three *A. willeyana *individuals relative to six other genes using quantitative real time PCR (qPCR). *Awi-Spherulin *is expressed on average 20 times higher than the previously characterized spherulite-forming gene *Astrosclerin *[7], and at almost 28% the level of *actin *(Figure 3), indicating it is a highly expressed gene. We then surveyed the relative synonymous codon usage (RSCU) of 181 *A. willeyana *evolutionarily conserved genes (Additional file 3), and compared these RSCU patterns with *Awi-Spherulin *and *Spherulin *homologs from 10 prokaryotes. The high level of *Awi-Spherulin *expression has clearly shaped its codon usage bias such that it is now more similar to eukaryotic genes that have no HGT ancestry (Figure 3). This result supports the hypothesis that the *Spherulin-*HGT event took place hundreds of millions of years ago. Intriguingly, *A. queenslandica *does not form any calcified structures, suggesting that the functions of the two poriferan Spherulins have diverged from each other. In support of this we could not detect any *Aqu-Spherulin *transcripts among 66,000+ *A. queenslandica *ESTs derived from 3 distinct larval cDNA libraries, suggesting that *Aqu-Spherulin *is not expressed during development.

While there are many examples of HGT between prokaryotes [20], well established examples of HGT from prokaryotic into metazoan organisms are relatively few, with some examples revealed to be artifacts of improper analysis [13,21-23]. When they are detected, prokaryotic to eukaryotic HGTs often reflect relatively recent events because commonly employed methods to detect them (phylogenetic incongruence and composition analyses) require the maintenance of a phylogenetic signal [14]. Indeed the only method that would detect the *Spherulin *HGT is a phyletic pattern analysis [24,25]. In this type of analysis the phyletic distribution of the gene of interest does not reflect the known evolutionary relationships of the species it is found in. The number of independent gene losses that must be invoked in order to account for such a distribution suggests that another explanation (HGT) should be considered. Our fortuitous identification of the HGT that established *Spherulin *in an ancestral poriferan genome highlights the fact that phylogenetic incongruence and composition based analyses may miss HGTs between clades of organisms that either have poor genomic resources, and/or represent HGTs that occurred in the distant past.

While the HGT we describe here may have been the result of a transfer of a sponge gene into a bacterial endobiont, this is extremely unlikely due to the phyletic distribution of *Spherulin *homologs throughout the Proteobacteria; *Spherulin *homologs are present in delta- and gamma-proteobacteria (Figure 2), and can also be detected in alpha-proteobacteria and enterobacteria. Such a distribution could only be possible if the sponge *Spherulin *gene had been horizontally transferred from an ancestral sponge into each of these distinct bacterial lineages independently. Alternatively, sponges would need to have been present at a time when the last common ancestor of these bacterial lineages existed, a scenario not supported by fossil or molecular data.

### The *Spherulin *gene likely supported the evolution of the *Astrosclera *biomineralization strategy

HGTs are thought to often involve the transfer of enzymes involved in metabolic processes [26, however see, 27 for an alternative view]. Given its similarity with sugar transporters, the modern and/or ancestral function of *Awi-*Spherulin suggests that the *Spherulin-*HGT falls within this category. It is tempting to speculate that *Awi-*Spherulin is functionally required to supply the energy necessary for the metabolically demanding process of biocalcification, however such a hypothesis awaits support by functional characterization of *Awi-*Spherulin. Unfortunately, *in vivo *manipulation of *Awi-Spherulin *expression or translation is currently not possible as methods to culture *A. willeyana *in the laboratory have not been established. Additionally there is no functional data available for any of the prokaryotic Spherulin homologs. Nonetheless, the presence of *Awi*-Spherulin protein in purified spherulites, and its high level of expression exclusively in spherulite forming cells is strong evidence that it plays a direct role in biocalcification in *A. willeyana*.

## Conclusions

Sponges are well known for the abundant bacterial communities they harbor [28], a condition that significantly increases the likelihood of HGTs [14]. We provide here the first example of a HGT event into a sponge genome from a prokaryote, which serves to highlight the intimacy of the sponge-bacteria relationship. The fact that *Awi-Spherulin *is most likely involved in skeletogenesis suggests that this HGT event may have contributed to the evolution of *A. willeyana*'s bodyplan. With the growing availability of metazoan genomes, and our understanding of the mechanisms that support the mobilization of genetic material, more instances of HGT into and between eukaryotic genomes will certainly be revealed. We predict that such events will often be associated with fundamental evolutionary changes to gene regulation, gene repertoire and morphological diversity.

## Methods

Spherulites were isolated from snap frozen *A. willeyana *individuals collected around Lizard Island (Great Barrier Reef, Australia) and during the Deep DownUnder Expedition (http://www.deepdownunder.de) at various reefs on the Queensland Plateau (Australia). A prominent band at 52 kDa was excised from a standard 12% SDS-PAGE gel and sequenced by Edman degradation. From this sequence a degenerate primer (5'-TGICCDATRTCIGGDATISWRTTRTADATICCIAC-3') was designed and used to isolate a 3' fragment from a cDNA library cloned into a λ TripleX vector (Clontech). 3' and 5' RACE fragments were subsequently isolated and assembled into a putative full length mRNA. The significant BLAST hit of *Awi-Spherulin *against *S. aurantiaca *was statistically tested and confirmed using PRSS3 [29]. WMISH was performed as previously described [7] using 1,234 bp DIG labeled sense and anti-sense probes. Comparative quantitative RT-PCR was performed using a Qiagen Rotor-Gene Q machine and SYBR green chemistry. Primer sequences to all genes are available in Additional file 4. For each *A. willeyana *individual *Spherulin *was set as the calibrator. A set of Spherulin protein homologs was downloaded from GenBank and aligned using ClustalW2 with gap opening and gap extension penalties set to 3 and 1.8 respectively (see Additional file 5 for the alignment). Phylogenetic analyses were conducted using MrBayes v. 3.1.2. Four runs each with 8 chains were run for 1.1 million generations at a temperature of 0.2. The first 25% of these trees were discarded as burnin. A correspondence analysis of relative synonymous codon usage was conducted using CodonW (http://sourceforge.net/projects/codonw/). *A. willeyana *CDSs derived from an EST collection were assembled and searched against NCBI's nr database in order to unambiguously identify correct reading frames and start and stop codons from highly conserved genes (minimum BLASTx e value 10^-6^). Sequences with high similarity to bacterial sequences were removed leaving a total of 181 *A. willeyana *CDSs (Additional file 3).

## Authors' contributions

DJJ designed experiments, conducted the WMISH and qPCR work, conducted the bioinformatic analyses and wrote the manuscript. GW conceived the study, guided initial phases of the project and drafted the manuscript. JR contributed analytical tools and reagents. LM isolated RACE fragments and provided technical support. All authors read and approved the final manuscript.

## Supplementary Material

Additional file 1 **The full-length sequence of *Awi-Spherulin***. The full-length sequence of *Awi-Spherulin *with annotations. Coding DNA sequence is uppercase. The signal sequence is highlighted grey, a 940 bp intron is highlighted red, a polypyrimidine tract is highlighted in white, two putative polyadenylation signals are boxed and the peptide recovered by Edman sequencing is highlighted green. Click here for file

Additional file 2 **A list of the Eukaryotic genomes exhaustively searched by BLAST for Spherulin homologs**. The following draft genome sequences were searched for the presence of Spherulin homologs using NCBI's genomic node-based BLAST tool. Click here for file

Additional file 3 **The *A. willeyana *genes used to asses codon usage bias**. These genes were collected from an in-house EST dataset and were used to establish the typical codon usage spread of *A. willeyana *genes. Against this background, the codon usage biases of Awi-*Spherulin *and *Spherulin *homologs form prokaryotes was plotted using CodonW. Click here for file

Additional file 4 **Primer sequences**. A list of primer sequences used to amplify the genes investigated in this study. Click here for file

Additional file 5 **Alignment of Spherulin homologs**. An alignment in FASTA format of Spherulin homologs from *A. willeyana, A. queenslandica *and 7 proteobacteria generated by ClustalW2 with gap opening and gap extension penalties set to 3 and 1.8 respectively. Click here for file
