# Supplementary material for: A horizontal gene transfer supported the evolution of an early metazoan biomineralization strategy
Source: BMC Evol Biol. 2011 Aug 12;11:238. doi: 10.1186/1471-2148-11-238 (PMC3163562; doi:10.1186/1471-2148-11-238)
Supplement: Additional file 1 — The full-length sequence of Awi-Spherulin. The full-length sequence of Awi-Spherulin with annotations. Coding DNA sequence is uppercase. The signal sequence is highlighted grey, a 940 bp intron is highlighted red, a polypyrimidine tract is highlighted in white, two putative polyadenylation signals are boxed and the peptide recovered by Edman sequencing is highlighted green. [file 1471-2148-11-238-S1.PDF]

Additional file 1. Annotated full-length sequence of *Awi-Spherulin.* Coding DNA sequence is uppercase. The signal sequence is highlighted grey, a 940bp intron is highlighted red, a polypyrimidine tract is highlighted in white, two putative polyadenylation signals are boxed and the peptide recovered by Edman sequencing is highlighted green.

```
   1 – aagtcaactcgctacagagtgtggggcttgctcgggcttgcctcgttcttacgttcagca –   60
  61 – gcaacctgaactgaccaagttgctaacgatcagtcgagcagaattaggcgacagtgacgg –  120
 121 – ccgtgtgatttcttgttacattcaggATGAACAGAGCAgtatccatcactgaacctgcat –  180
   1 –                           M  N  R  A                          –    4
 181 – attcacaagctctgcattgtttattgtaaactatataactgtatatcgaaataaataatc –  240
 241 – ctgtgacaatcttttctggtacgaatgcttacatgcaaatagtgcaaatagtctaacatc –  300
 301 – gtatagcggaaatattttgcgataaacaatttgttttgctgaagacctgcccatggcagc –  360
 361 – agatggcgcttggaagtgagatattagagtaggactgtgctacgcaaagcgtggagtcga –  420
 421 – acacacgcacctttaaatagcctttttcaaagcttaatccaactgttttccgcgtatgt –  480
 481 – caaatattactattcgcgtttacagccttccagtttttgatcgattacattatgcagcca –  540
 541 – ggcgttcaaaacctggagagttggacgtctgaaaatgaggccagattattaccctgtcgt –  600
 601 – cgttcctagaaaaccgcccatcgcttgcagtagtggtcagtgttcatttggctcgtaaca –  660
 661 – tctcacaggcaagatcttgatcgctttacagtcggtactttaagcggaaatgtacgccgc –  720
 721 – tgatgtagctgcatatgagaactttttactactcatatataagactattgagactgctg –  780
 781 – cgttgaggagactgctgacagtaaaattataatttatagcaggttatgaatatgaattta –  840
 841 – aactgaatggcaaaaaagtttaaccactacatccgctatagttcaagatctatatgccac –  900
 901 – tgcatcatacttatagattattagatatcgaacgtctcacagtaaactcatttagtcgga –  960
 961 – ttgggacattgtcctgagagcccaggaaattgtacaacatatatagtgggcttatccgat – 1020
1021 – gcaaactgtgattctccacaataagtacgtagattgtatgtcctcatgcacttattcctt – 1080
1081 – tcatttcctccaacccagATCCAGATAGCAGGTTTGCTTTTCATACAGTTGGTCAGCCTT – 1140
   5 –                   I  Q  I  A  G  L  L  F  I  Q  L  V  S  L   –   18
1141 – AGTTCTGCTGCTGTGCAACTAAGAGTTGGAATTTACAATTCAATTCCTGATATTGGACAG – 1200
  19 – S  S  A  A  V  Q  L  R  V  G  I  Y  N  S  I  P  D  I  G  Q   –   38
1201 – GATAATCTGACGTCCTACAAAGGTCTGATAGAGGGAGGTTTCAACAATGCTGCCCATACC – 1260
  39 – D  N  L  T  S  Y  K  G  L  I  E  G  G  F  N  N  A  A  H  T   –   58
1261 – GTTGATGCTGTTGTAGATACAACTGAATATGATCCTTATGGAGATCTAACAACATATCTT – 1320
  59 – V  D  A  V  V  D  T  T  E  Y  D  P  Y  G  D  L  T  T  Y  L   –   78
1321 – TCTGAGGATGGTTTTGACATGATTGAAATGGACACAGCCAACTTAAAAGAGGTGGTTGAG – 1380
  79 – S  E  D  G  F  D  M  I  E  M  D  T  A  N  L  K  E  V  V  E   –   98
1381 – GATGACCTCATCATAGATATACCAACAAATCTTCCAGAGAATATCATGCCAGCCGCTGTA – 1440
  99 – D  D  L  I  I  D  I  P  T  N  L  P  E  N  I  M  P  A  A  V   –  118
1441 – GGAGCTGCAGCAATAAACGGAAAACTGTATGCTTACCCCACGCTGCTCTGTGGCAACTTC – 1500
 119 – G  A  A  A  I  N  G  K  L  Y  A  Y  P  T  L  L  C  G  N  F   –  138
1501 – CTTATTGGACTTGTTCCACCTGGCAATGAACAAAATTGCCCACTGAGAAATGCTCGAGTA – 1560
 139 – L  I  G  L  V  P  P  G  N  E  Q  N  C  P  L  R  N  A  R  V   –  158
1561 – GATTACAATGCTTTCTATGAAACCATGGAGAACTGCAAACAAAACGTTGGTGGAGACTGG – 1620
 159 – D  Y  N  A  F  Y  E  T  M  E  N  C  K  Q  N  V  G  G  D  W   –  178
1621 – AGGAGAATACTTGGTGGAAAAATGAACGATGATTATGGTTGGTACTTACCCTATCTCTAC – 1680
 179 – R  R  I  L  G  G  K  M  N  D  D  Y  G  W  Y  L  P  Y  L  Y   –  198
1681 – CTTGATGGTTACATTGATATCCATGGTAGGGAATCAGTTGATAAAGCCGTTGATGAGGTC – 1740
 199 – L  D  G  Y  I  D  I  H  G  R  E  S  V  D  K  A  V  D  E  V   –  218
1741 – ATGAGAGGAGTTGTGGACCCAAAAGTGTGTGAAAGGCTTAGTTGGTACATCGGTTGCTGT – 1800
 219 – M  R  G  V  V  D  P  K  V  C  E  R  L  S  W  Y  I  G  C  C   –  238
1801 – GATGACAAAACAGGTCAGGTTGGAAACAAGTGCTATGAAAATTTTATAGGAAGCTATGTA – 1860
 239 – D  D  K  T  G  Q  V  G  N  K  C  Y  E  N  F  I  G  S  Y  V   –  258
1861 – AATGACAGTGATAATCTGTACCCTGATATAATAAATGGGGAAACAGCTTTTTACTTTGGC – 1920
 259 – N  D  S  D  N  L  Y  P  D  I  I  N  G  E  T  A  F  Y  F  G   –  278
1921 – TTCTCTGAAAAGGTTGCTCAAGTTGAATTTGATCGAAATTCTTATGCTGCTATCTCTGGA – 1980
 279 – F  S  E  K  V  A  Q  V  E  F  D  R  N  S  Y  A  A  I  S  G   –  298
1981 – CCACTGGGAGAAATCAACAATCTGCTCCAGTTTACAGATGCACTGGTCATCAACAAGGCA – 2040
 299 – P  L  G  E  I  N  N  L  L  Q  F  T  D  A  L  V  I  N  K  A   –  318
2041 – CGCTGGAATGCAGCAAATGATGAGAAGAGGAATGCCATAATTGATTTTGTGAACTACTTT – 2100
 319 – R  W  N  A  A  N  D  E  K  R  N  A  I  I  D  F  V  N  Y  F   –  338
2101 – CTAAACAATAATCTTCGTGAAGATATTGCAATGGGAGTTGACCTCAACCCACCTCAAGTC – 2160
 339 – L  N  N  N  L  R  E  D  I  A  M  G  V  D  L  N  P  P  Q  V   –  358
2161 – CGCTACCTCCTCCAATCTACTGAGACATTCTATCAGAATACAACTGACCTCATTTATCAA – 2220
 359 – R  Y  L  L  Q  S  T  E  T  F  Y  Q  N  T  T  D  L  I  Y  Q   –  378
2221 – GATCTATTTTGGTCACTTCAGAGAGCTGTGGCCGCACCATCTCTTACTAGCTATCAGAAA – 2280
 379 – D  L  F  W  S  L  Q  R  A  V  A  A  P  S  L  T  S  Y  Q  K   –  398
2281 – GTGACTATGGAAGCAAATCTTGAAAGTTTGTGCATAAAATTTCCTCAAAGCAAGAAAATG – 2340
 399 – V  T  M  E  A  N  L  E  S  L  C  I  K  F  P  Q  S  K  K  M   –  418
2341 – AGGAAATTTAAACAAGAACTGtaatgtttactaagtcttgaacatgtatctgtttgtagg – 2400
 419 – R  K  F  K  Q  E  L  *                                         –  425
2401 – ttgactgcttggctagttgtaaactttgtgtaatcaccttgatttatat[aattaa]tgaat – 2460
2461 – gattgta[aatcaa]tttggttgtctacatagtggttaccctggttgcagacagccaaaaaa – 2520
2521 – aaaaaaaaaaaaaaaaaaaa                                         – 2580
```
