# Supplementary material for: A horizontal gene transfer supported the evolution of an early metazoan biomineralization strategy
Source: BMC Evol Biol. 2011 Aug 12;11:238. doi: 10.1186/1471-2148-11-238 (PMC3163562; doi:10.1186/1471-2148-11-238)
Supplement: Additional file 2 — A list of the Eukaryotic genomes exhaustively searched by BLAST for Spherulin homologs. The following draft genome sequences were searched for the presence of Spherulin homologs using NCBI's genomic node-based BLAST tool. [file 1471-2148-11-238-S2.DOC]

**Additional file 2. A list of the Eukaryotic genomes exhaustively searched by BLAST for Spherulin homologs.** The following draft genome sequences were searched for the presence of Spherulin homologs using NCBI’s genomic node-based BLAST tool.

**Eukaryota**

**Apicomplexa**

Ascogregarina taiwanensis

Babesia bovis

Cryptosporidium hominis

Cryptosporidium muris RN66

Cryptosporidium parvum Iowa II

Eimeria tenella str. Houghton

Plasmodium berghei

Plasmodium chabaudi chabaudi

Plasmodium falciparum

Plasmodium knowlesi strain H

Plasmodium vivax

Plasmodium yoelii yoelii

Theileria annulata strain Ankara

Theileria parva

Toxoplasma gondii

**Dictyosteliida**

Dictyostelium discoideum AX4

Polysphondylium pallidum PN500

**Diplomonadida**

Giardia intestinalis

**Entamoebidae**

Entamoeba dispar SAW760

Entamoeba histolytica HM-1:IMSS

Entamoeba invadens IP1

**Fungi/Metazoa group**

**Fungi**

**Ascomycota**

**Pezizomycotina**

Ajellomyces capsulatus

Ajellomyces dermatitidis

Alternaria brassicicola ATCC 96836

Arthroderma benhamiae CBS 112371

Arthroderma gypseum CBS 118893

Arthroderma otae CBS 113480

Ascosphaera apis USDA-ARSEF 7405

Aspergillus clavatus NRRL 1

Aspergillus flavus NRRL3357

Aspergillus nidulans FGSC A4

Aspergillus niger CBS 513.88

Aspergillus terreus

Blumeria graminis f. sp. hordei DH14

Botryotinia fuckeliana B05.10

Chaetomium globosum CBS 148.51

Coccidioides immitis

Coccidioides posadasii

Epichloe festucae E2368

Fusarium oxysporum f. sp. lycopersici 4287

Gaeumannomyces graminis var. tritici R3-111a-1

Geomyces destructans 20631-21

Gibberella moniliformis 7600

Gibberella zeae PH-1

Glomerella graminicola M1.001

Grosmannia clavigera

Magnaporthe oryzae 70-15

Nectria haematococca mpVI 77-13-4

Neosartorya fischeri NRRL 181

Neosartorya fumigata

Neurospora crassa OR74A

Paracoccidioides brasiliensis

Penicillium chrysogenum Wisconsin 54-1255

Penicillium marneffei ATCC 18224

Phaeosphaeria nodorum SN15

Podospora anserina S mat+

Pyrenophora teres f. teres 0-1

Pyrenophora tritici-repentis Pt-1C-BFP

Sclerotinia sclerotiorum 1980 UF-70

Sordaria macrospora

Talaromyces stipitatus ATCC 10500

Trichoderma atroviride IMI 206040

Trichoderma reesei QM6a

Trichoderma virens Gv29-8

Trichophyton equinum CBS 127.97

Trichophyton rubrum CBS 118892

Trichophyton tonsurans CBS 112818

Trichophyton verrucosum HKI 0517

Tuber melanosporum

Uncinocarpus reesii 1704

Verticillium albo-atrum VaMs.102

Verticillium dahliae VdLs.17

**Saccharomycotina**

Ashbya gossypii ATCC 10895

Candida albicans

Candida dubliniensis CD36

Candida glabrata CBS 138

Candida parapsilosis CDC317

Candida tropicalis MYA-3404

Clavispora lusitaniae ATCC 42720

Debaryomyces hansenii CBS767

Kluyveromyces aestuarii ATCC 18862

Kluyveromyces lactis NRRL Y-1140

Kluyveromyces wickerhamii UCD 54-210

Lachancea kluyveri NRRL Y-12651

Lachancea thermotolerans CBS 6340

Lachancea waltii NCYC 2644

Lodderomyces elongisporus NRRL YB-4239

Meyerozyma guilliermondii ATCC 6260

Pichia pastoris

Pichia pastoris DSMZ 70382

Saccharomyces bayanus

Saccharomyces castellii NRRL Y-12630

Saccharomyces cerevisiae

Saccharomyces kudriavzevii IFO 1802

Saccharomyces mikatae

Saccharomyces paradoxus NRRL Y-17217

Saccharomyces pastorianus Weihenstephan 34/70

Scheffersomyces stipitis CBS 6054

Vanderwaltozyma polyspora DSM 70294

Wickerhamomyces anomalus NRRL Y-366

Yarrowia lipolytica CLIB122

Zygosaccharomyces rouxii CBS 732

**Schizosaccharomycetes**

Schizosaccharomyces cryophilus NRRL Y-48691

Schizosaccharomyces japonicus yFS275

Schizosaccharomyces octosporus yFS286

Schizosaccharomyces pombe

**Basidiomycota**

Coprinopsis cinerea okayama7#130

Cryptococcus bacillisporus R265

Filobasidiella neoformans

Laccaria bicolor S238N-H82

Malassezia globosa CBS 7966

Malassezia restricta CBS 7877

Microbotryum violaceum p1A1 Lamole

Mixia osmundae IAM 14324

Moniliophthora perniciosa FA553

Phanerochaete chrysosporium RP-78

Postia placenta Mad-698-R

Puccinia graminis f. sp. tritici CRL 75-36-700-3

Puccinia triticina 1-1 BBBD Race 1

Schizophyllum commune H4-8

Ustilago maydis 521

**Microsporidia**

Encephalitozoon cuniculi GB-M1

Encephalitozoon intestinalis ATCC 50506

Enterocytozoon bieneusi H348

Nematocida parisii ERTm1

Nosema ceranae BRL01

Octosporea bayeri OER-3-3

**Metazoa**

**Acoelomata**

**Trematoda**

Schistosoma japonicum

Schistosoma mansoni

**Coelomata**

**Arthropoda**

Acyrthosiphon pisum

Aedes aegypti

Anopheles darlingi

Anopheles gambiae

Apis florea

Apis mellifera

Atta cephalotes

Bombus terrestris

Bombyx mori

Camponotus floridanus

Culex quinquefasciatus

Daphnia pulex

Drosophila ananassae

Drosophila erecta

Drosophila grimshawi

Drosophila melanogaster

Drosophila mojavensis

Drosophila persimilis

Drosophila pseudoobscura

Drosophila pseudoobscura pseudoobscura

Drosophila sechellia

Drosophila simulans

Drosophila virilis

Drosophila willistoni

Drosophila yakuba

Harpegnathos saltator

Ixodes scapularis

Lepeophtheirus salmonis

Mayetiola destructor

Nasonia giraulti

Nasonia longicornis

Nasonia vitripennis

Pediculus humanus corporis

Rhipicephalus microplus

Rhodnius prolixus

Tribolium castaneum

Varroa destructor

**Chordata**

**Mammals**

Ailuropoda melanoleuca

Bos taurus

Bubalus bubalis

Callithrix jacchus

Canis lupus

Canis lupus familiaris

Cavia porcellus

Choloepus hoffmanni

Dasypus novemcinctus

Dipodomys ordii

Echinops telfairi

Equus caballus

Erinaceus europaeus

Felis catus

Gorilla gorilla

Homo sapiens

Lama pacos

Loxodonta africana

Macaca mulatta

Macropus eugenii

Microcebus murinus

Monodelphis domestica

Mus musculus

Myotis lucifugus

Nomascus leucogenys

Ochotona princeps

Ornithorhynchus anatinus

Oryctolagus cuniculus

Otolemur garnettii

Ovis aries

Pan troglodytes

Pongo abelii

Procavia capensis

Pteropus vampyrus

Rattus norvegicus

Sorex araneus

Spermophilus tridecemlineatus

Sus scrofa

Tarsius syrichta

Tupaia belangeri

Tursiops truncatus

**Others**

Anolis carolinensis

Branchiostoma floridae

Callorhinchus milii

Ciona intestinalis

Ciona savignyi

Danio rerio

Dicentrarchus labrax

Gallus gallus

Gasterosteus aculeatus

Labeotropheus fuelleborni

Maylandia zebra

Mchenga conophoros

Melanochromis auratus

Meleagris gallopavo

Nothobranchius furzeri

Nothobranchius kuhntae

Oikopleura dioica

Oryzias latipes

Rhamphochromis esox

Taeniopygia guttata

Takifugu rubripes

Tetraodon nigroviridis

Xenopus (Silurana) tropicalis

**Mollusca**

Aplysia californica

Lottia gigantea

**Hemichordata**

Saccoglossus kowalevskii

**Echinodermata**

Strongylocentrotus purpuratus

**Annelida**

Capitella teleta

Helobdella robusta

**Cnidaria**

Nematostella vectensis

**Placozoa**

Trichoplax adhaerens

**Choanoflagellata**

Monosiga brevicolis

**Nematoda**

Brugia malayi

Caenorhabditis brenneri

Caenorhabditis briggsae

Caenorhabditis briggsae AF16

Caenorhabditis elegans

Caenorhabditis japonica

Caenorhabditis remanei

Caenorhabditis sp. PS1010

Heterodera glycines

Loa loa

Meloidogyne hapla

Meloidogyne incognita

Onchocerca volvulus

Pristionchus pacificus

Trichinella spiralis

Wuchereria bancrofti

**Kinetoplastida**

Leishmania braziliensis MHOM/BR/75/M2904

Leishmania infantum JPCM5

Leishmania major strain Friedlin

Trypanosoma brucei

Trypanosoma cruzi

**Others**

Bigelowiella natans

Guillardia theta

Hemiselmis andersenii

Naegleria gruberi

Paramecium tetraurelia

Perkinsus marinus ATCC 50983

Tetrahymena thermophila SB210

Thecamonas trahens ATCC 50062

Trichomonas vaginalis G3

**Stramenopiles**

Blastocystis hominis

Ectocarpus siliculosus

Hyaloperonospora arabidopsidis Emoy2

Phaeodactylum tricornutum CCAP 1055/1

Phytophthora infestans T30-4

Phytophthora ramorum

Phytophthora sojae

Pythium ultimum DAOM BR144

Saprolegnia parasitica CBS 223.65

Thalassiosira pseudonana CCMP1335
