# Supplementary material for: A horizontal gene transfer supported the evolution of an early metazoan biomineralization strategy
Source: BMC Evol Biol. 2011 Aug 12;11:238. doi: 10.1186/1471-2148-11-238 (PMC3163562; doi:10.1186/1471-2148-11-238)
Supplement: Additional file 3 — The A. willeyana genes used to asses codon usage bias. These genes were collected from an in-house EST dataset and were used to establish the typical codon usage spread of A. willeyana genes. Against this background, the codon usage biases of Awi-Spherulin and Spherulin homologs form prokaryotes was plotted using CodonW. [file 1471-2148-11-238-S3.DOC]

**Additional file 3.** **The *A. willeyana* genes used to asses codon usage bias.** These genes were collected from an in–house EST dataset and were used to establish the typical codon usage spread of *A. willeyana* genes. Against this background, the codon usage biases of Awi-*Spherulin* and *Spherulin* homologs form prokaryotes was plotted using CodonW.

DMPC13981222 Actin

DMPC13961823 beta catenin

DMPC13962138 26S protease subunit

DMPC13961763 histone-arginine methyltransferase

DMPC13961845 Cathepsin C

DMPC13961472 Clathrin light polypeptide

DMPC13961965 Dynein light chain

DMPC13961464 carnitine O-palmitoyltransferase

DMPC13961757 Cathepsin L

DMPC13961878 Calmodulin

DMPC13961595 actin-related protein 2/3

DMPC13962046 ADP-ribosylation factor

DMPC13980713 hypothetical protein

DMPC13961567 aspartic proteinase

DMPC13961777 ATP synthase, H+ transporting

DMPC13962115 Astrosclerin1

DMPC13961575 Cyclophilin

DMPC13961808 2-dehydro-3-deoxyglucarate aldolase

DMPC13962096 40S ribosomal protein S21

DMPC13961796 40S ribosomal protein S24

DMPC2083076 40S ribosomal protein S25

DMPC13961344 60S acidic ribosomal protein P0

DMPC13981225 60S acidic ribosomal protein P1

DMPC13961368 60S ribosomal protein L37-A

DMPC2083037 60S ribosomal protein L44

DMPC13961470 60S ribosomal protein L7a

DMPC13980397 aarF domain containing kinase 5

DMPC13962041 acetoacetyl-CoA synthetase

DMPC13962082 acetoacetyl-CoA thiolase

DMPC13961595 actin related protein 2/3 complex subunit 1

DMPC13961862 actin related protein 3

DMPC13961373 Actin

DMPC13962002 Gelsolin actin-modulator

DMPC13961779 activator of G-protein signal

DMPC13961862 Actr3 protein

DMPC13961982 acyl-CoA oxidase type 1

DMPC13961799 adenosine deaminase

DMPC13962046 ADP-ribosylation factor 4

DMPC13961979 aggregation factor protein 3

DMPC13961379 aldehyde reductase

DMPC13961339 Ankrd45 protein

DMPC13961860 Annexin A4

DMPC13961895 APC-like

DMPC13961447 apoptosis-linked gene

DMPC2083126 similar to vibrator

DMPC13961529 hypothetical protein

DMPC13962064 galactosamine

DMPC13980463 Eukaryotic translation initiation factor 5A-1

DMPC13961759 lipopolysaccharide-induced TNF factor

DMPC13961623 predicted protein

DMPC13961517 thioredoxin

DMPC13962003 Epididymal secretory protein

DMPC13961958 cytidylate kinase

DMPC13961506 ribosomal protein L3

DMPC13962117 testis-specific gene A2

DMPC13961898 similar to polymerase

DMPC13962091 peptidylprolyl isomerase B

DMPC13961528 proteasome 26S non-ATPase subunit 7

DMPC13962008 interferon-gamma-inducible lysosomal thiol

DMPC13962057 DC2 protein-like

DMPC2083107 ankyrin repeat-containing protein

DMPC13961423 cyclic AMP-regulated protein like

DMPC2083153 DNA-directed RNA polymerase II subunit

DMPC13961467 tropomyosin

DMPC13981202 actin related protein 2/3

DMPC13980696 DnaJ

DMPC13961413 hypothetical protein

DMPC13961652 Actin-related protein 2-A

DMPC13961788 acyl-Coenzyme A dehydrogenase

DMPC13961557 novel protein

DMPC2083212 DSBA thioredoxin oxdioreductase

DMPC13962077 enhancer of yellow 2

DMPC13961999 ribosomal protein L40

DMPC13961674 predicted protein

DMPC13961876 60S ribosomal protein L37a

DMPC13961763 histone-arginine methyltransferase

DMPC13961544 hypothetical protein

DMPC13961923 hypothetical protein

DMPC13962108 predicted protein

DMPC13980385 14-3-3b protein

DMPC13961630 hypothetical protein

DMPC2083052 similar to Ras-related

DMPC13961354 Glutathione S-transferase domain

DMPC13961755 cytochrome c oxidase subunit VIb

DMPC13961932 ribosomal protein L22e

DMPC13980426 nonmuscle myosin II regulatory light chain

DMPC13961573 mitogen-activated protein kinase

DMPC13962105 TAF12 RNA polymerase II

DMPC13980422 predicted protein

DMPC13962054 hypothetical protein Krac_1408

DMPC13981011 40S ribosomal protein S23

DMPC2083158 HIG1 hypoxia inducible domain

DMPC13961503 ankyrin repeat protein

DMPC13961783 G2/mitotic-specific cyclin B1

DMPC13961526 FK506-binding protein

DMPC13961575 peptidyl-prolyl cis-trans isomerase F

DMPC13961543 cytochrome c oxidase subunit IV

DMPC13981297 predicted protein

DMPC13961368 60S ribosomal protein L37-A

DMPC2083076 S25e ribosomal protein

DMPC13961784 ribosomal protein L26

DMPC13961490 KN motif and ankyrin repeat domains

DMPC2083225 Glutaredoxin-related protein

DMPC13961806 forkhead foxD

DMPC13962154 hypothetical protein

DMPC13961381 predicted protein

DMPC13981102 predicted protein

DMPC13962133 putative 40S ribosomal protein S28

DMPC13961472 clathrin, light polypeptide

DMPC13980882 conserved hypothetical protein

DMPC13980409 putative selenoprotein X

DMPC13961796 40S ribosomal protein S24

DMPC13961430 COMM domain-containing protein 2

DMPC13962147 ribosome production factor

DMPC13961965 hypothetical protein

DMPC13961984 ubiquitin-conjugating enzyme E2 J2

DMPC13961878 similar to calmodulin 2

DMPC13961693 predicted protein

DMPC2083173 Protein C20orf11

DMPC13961339 predicted protein

DMPC13961714 ATP synthase, H+ transporting

DMPC13961445 predicted protein

DMPC2083118 hypothetical protein

DMPC13961790 hypothetical protein

DMPC2083037 60S ribosomal protein L44

DMPC13961869 predicted protein

DMPC13962087 predicted protein

DMPC13981050 hypothetical protein

DMPC13961967 mps one binder kinase activator

DMPC13961647 ribosomal protein S15e

DMPC13961420 prostaglandin D2 synthase

DMPC13961979 aggregation factor protein 3

DMPC2083215 hypothetical protein

DMPC13980393 astrosclerin-2

DMPC13961734 hypothetical protein

DMPC13961907 vascular endothelial growth factor C

DMPC13961668 Cytochrome c oxidase subunit 5A

DMPC2083068 zinc finger (MYND)-13

DMPC13961901 similar to membrane protein

DMPC13961944 U6 snRNA-associated Sm-like protein

DMPC2083192 protein lin-7 homolog C

DMPC13961769 hypothetical protein

DMPC13961498 Arf10

DMPC13961646 similar to Cytochrome c proxima

DMPC2083063 PREDICTED

DMPC13980979 polyubiquitin

DMPC13961672 Ras-related protein Rab-2

DMPC13962033 predicted protein

DMPC13961538 hypothetical protein

DMPC13961435 ubiquitin protein ligase

DMPC2083084 hypothetical protein

DMPC13980828 hypothetical protein

DMPC13961383 predicted protein

DMPC13961781 hypothetical protein

DMPC2083090 hypothetical protein

DMPC13962032 predicted protein

DMPC2083066 40S ribosomal protein S16

DMPC13961778 glutamate-cysteine ligase catalytic subunit

DMPC13961531 predicted protein

DMPC13962048 Eif4a1b protein

DMPC13961888 predicted protein

DMPC13961733 hypothetical protein

DMPC13961519 60S ribosomal protein L38

DMPC13961946 prefoldin subunit 4-like

DMPC13962125 hypothetical protein

DMPC13962030 conserved hypothetical protein

DMPC13961530 predicted protein

DMPC13961666 Rab35

DMPC13980580 novel protein

DMPC13961550 receptor expression-enhancing protein

DMPC13961857 40S ribosomal protein S18

DMPC13980800 lectin

DMPC2083142 Proteasome subunit beta type-7

DMPC13961926 phosphatase and actin regulator

DMPC13980854 Transmembrane emp24 domain

DMPC13961924 Histone H3.3

DMPC13961446 cystatin-A

DMPC13961978 predicted protein

DMPC13961775 predicted protein

DMPC13961885 ribosomal protein rpl13a

DMPC13962029 ARV1
