# Supplementary material for: A horizontal gene transfer supported the evolution of an early metazoan biomineralization strategy
Source: BMC Evol Biol. 2011 Aug 12;11:238. doi: 10.1186/1471-2148-11-238 (PMC3163562; doi:10.1186/1471-2148-11-238)
Supplement: Additional file 4 — Primer sequences. A list of primer sequences used to amplify the genes investigated in this study. [file 1471-2148-11-238-S4.DOC]

**Additional file 4.** Primer sequences used in this study.

| **Gene** | **Primer sequence (5' to 3')** | **Product size (bp)** |
| --- | --- | --- |
| Actin forward | CCGGGTTCGCCGGGGATGAT | 248 |
| Actin reverse | GTGCTCCTCGGGCGCAACA |
| Cyclin forward | TCCCATCCACTTTCTGCGCCGCTAC | 154 |
| Cyclin reverse | TGCCAGGAGGAAAGCGCCTGCTGCTC |
| Spherulin forward | GGCACGCTGGAATGCAGCAAATGA | 219 |
| Spherulin reverse | GCGGCCACAGCTCTCTGAAGTGACCA |
| β-Catenin forward | GCCGCCACCCGAATGCCGAGATGG | 176 |
| β-Catenin reverse | GCCCTGCTCTCGGAGCACTGTGTGGT |
| ATPase V_0_ forward | TGTGACCCTGTCCGGACTTTTGTTCCA | 151 |
| ATPase V_0_ reverse | TAACGGCGAAAAACGCGGCATA |
| Acyl-CoA forward | CGGCTGTTCTCAGCATCGCCTCGATTG | 236 |
| Acyl-CoA reverse | TCCAGGCTACTCAAGCCCACACCACCA |
| Astrosclerin forward | AGAAGGGTGGAGAGTGATGAGGA | 117 |
| Astrosclerin reverse | GTCTTCGCCAYTATCAACATCTGG |
