# Supplementary material for: A horizontal gene transfer supported the evolution of an early metazoan biomineralization strategy
Source: BMC Evol Biol. 2011 Aug 12;11:238. doi: 10.1186/1471-2148-11-238 (PMC3163562; doi:10.1186/1471-2148-11-238)
Supplement: Additional file 5 — Alignment of Spherulin homologs. An alignment in FASTA format of Spherulin homologs from A. willeyana, A. queenslandica and 7 proteobacteria generated by ClustalW2 with gap opening and gap extension penalties set to 3 and 1.8 respectively. [file 1471-2148-11-238-S5.DOC]

**Additional file 5.** An alignment in FASTA format of Spherulin homologs from *A. willeyana*, *A. queenslandica* and 7 proteobacteria generated by ClustalW2 with gap opening and gap extension penalties set to 3 and 1.8 respectively.

>Awi_Spherulin

------------------------------------------------------------

-----------------MNRAIQI--AGLLFIQLVSL--SS---------------AAVQ

LRVGIYNSIPDIGQDNLTSYKGLIEGGFNNA--AHTVDA-----------VVDTTEYD-P

YGDLTTYLSED-G---FDMIEMDTANLKEVVEDDLIIDIP--TNLP--ENIMPAAVGAA-

---AINGKLY-AYPTLLCGNFLIGLVPPGNEQNCPLRNARVDYNAFYETMENCKQNVGG-

-DWRRILGGKMNDDYGWYLPYLYLDGYIDIHGRESVDKAVDEVMRGVVDPKVCERLSWYI

GCC-DDKTG-QVGNKCYENFIGSYVNDSD-NLYPDIINGETAFYFGFSEKVA--QVERDS

-DRNSY--AAISGPLGEINNLLQFTDALVINKARWNAANDEKRNAIIDFVNYFLNNNLRE

DIAMGVDL-NPPQV-RYLLQSTETFY---QNTTDLIYQDLFWSLQRAVAAPSLTSYQKVT

MEANLESLCIKFPQSKKMRKFKQEL-----------------------------------

----------

>Aqu_Spherulin

------------------------------------------------------------

--------------------SVKY--FALIFACLAG---AA---------------IAVD

LKVGIYNSIPDIGDDDLASYENLIQDGYTAAYPSNTVDA-----------VVNSSLYD-P

YGNLEEYLHYD-G---FDMLEIDTISLPGLVEKGLIVPVDDLVYVPTWNDIFPEALDAV-

---QYEDTYY-AYPTLLCGNFLIGLSP-ATSGNCDLESGRYNYDNFSSILTQCEGLLSSY

PTYERLLGGKMNDIYGYYLPDMYVDGYIDMYGSQKAQEAVDNVLAGDIDMSLCSRMTNYV

GGC-SDETGSPPNNKCFYKYADSYVEESD-NIYTDITNKKTMLYFGFSEKLA--QIKKDN

PGIVAY--AAISAPLGDSGYLLQYTDALVVSASSWNSADNEKKAAMIDFITFFTSQSLRE

SILFGEDL-SPQAT-RYLLQANKQVYSIKEATSDPIIVDLYWALQRGVHT----------

------------------------------------------------------------

----------

>Sau DW4/3-1 gi|115377316|ref|ZP_01464524.1| conserved hypothetical protein d-proteobacteria [Stigmatella aurantiaca DW4/3-1]

------------------------------------------------------------

---MWCH-SMKITLKWGFIAGAVL--STLVGCSDEDP--EPVP-------------EKTQ

LRVPLYPYIPDAAGDQLQAMAARIESEFEQAHPEVDLIV---------NPSCFNDDFY-D

PEALARSLKGE-GECAYDVVETDTVILRELVALNAVRPWP---RLPQNIDWHPAGLAAS-

---RQQQSTY-GVPHWLCGDFIISRDE-------SVRQAR-TESALRHALAGLDTPKPD-

------MAVNLLG--SWNLPALYLDAWADRNGSANVASAVTTSNYDSVALQS---LRSFV

QTC--QS-AGA--NPCIDGTY-DQDENFD-LPATLFATGQVDATMGYSERLH-VIIRNLP

AGQSASDLKISSAPLAEGSHPILFTDSYFLGTR--CTG--ACEQAALAFVDYMSQPSTFE

WILMSEDAPAGTRVPRYLLPATLDSYATPKLQADPFYPVLNVESREGGPFPN-GGLLNIR

HQ--MRDDILTAITSEG-------------------------------------------

----------

>Sau DW4/3-1 gi|115375541|ref|ZP_01462799.1| hypothetical protein STIAU_8839 protein d-proteobacteria [Stigmatella aurantiaca DW4/3-1]

------------------------------------------------------------

---MSRL-AQATCL-YTLALCMGA--CAPAVH--AVR--QP-AA------------PPRP

FKAVLFPYIPDSANDQFASLIQTLTKNFKEQHPDIDLTI---------VMDQNMDLYDLS

DGGTLNQLLGP-APAAAQVVEVDTLLLGSLVTKNWIGPV----SMGNP-GVLETAWNAA-

---TIDNTAY-GIPTYLCSNVVYSRSA-------AIKAAT-GGSSLFSILTQMDPSKAP-

------LVANYKG--SWTLPGTYVDAWADTNP-GALTPAYNLP-VDPATMAV---FKPIV

SSCAKLSPTSTPSNPCLDG---QLK-KPG-AAAQVFATQKANGFMGYTEELF--NILSNS

-G-PLPPLTAISAPLGVGTRPVIFVDALVFNSN--CTG--PCLADAQAFAAFMSDTRVRS

LIAFSQDA-PKVSIPRYLLQANKAFYEVQPAASDAMYQQFLPIVHSAQPFPN-QGFPEHK

TA--LGDAVQQALTPSSVSFLFPSGGPLTEGRTSRAGAAPEPAGNTPGSTRRSGPRTAGP

HRSSAPSTLH

>Hoc DSM 14365 gi|262194628|ref|YP_003265837.1| putative lipoprotein d-proteobacteria [Haliangium ochraceum DSM 14365]

------------------------------------------------------------

---------MKIS-QFSAILALSL--CTQA-CGEMNPPAEPQPP------------TQTT

LRVSLFPWIPEA-----ESFFAWIEEDFERQHPDIDLIVRAVKKSHDWEPEYVADLSY-E

YEQTAEALTGD-GADAQDLVEVDTMLLGWLHSRDAIVPF----EVGDR-DYLPFAQQAV-

---SLGGEVY-GAPHWTCGYFVISEDP-------AIRQAA-DRAALLETLAARETDAVD-

------LVGDLDG--SWDSVMVYVDALHDGEPERDLVTALDEELIDPAVAES---FSAIG

AAC--TK-DGV--NGC------DSD-GVD-V----FARGEADALIGYSERLN-PILAD--

ADRSVGELHVASAPLGDGDHPVLFTDALVLSPL--CAE--RCREAAQQFAAYYNSDEVFE

TALLARDV-GDDAVPRYLLPATASAFETEGVAAERLYGELRTEIEGAVPYPI-TGVPEAR

ARGSIRAQIQTALGISP-------------------------------------------

----------

>Mxa DK1622 gi|108757766|ref|YP_632691.1| putative lipoprotein d-proteobacteria [Myxococcus xanthus DK 1622]

-----------------------------------------------------------M

IP-LPPLRGAAPSIHWSHTMQWRALPCVLALSLGACS--DPEPE------------APRP

LKVVLFPYIPDSAGDGFASLKQRLEADFEREHSDIDVDI---------VFDAKLDLYDLD

DGGTLNQLLGP-GAGAAQVVEIDTLIMGELVSKGWVQPV----ALEAG-AAHPAAEEAV-

---SIAGQSY-GVPTYLCSYVVYSNSP-------YLSSAT-DGDSLVQILTDVAPGMRP-

------LAANYSG--SWTLPSSYLDAWADTNPTGVLSQALSLP-LDASALAS---FEDVV

KSC-ELE-AGV--NPCLDG---TYA-DSS-LAEEAFALGQANGFMGYTERLF--FVRKAS

PGMALP--EVISVPLGTGSAPAVFVDALVVSAE--CTG--TCAEDAHAFTDFMKDPDVRS

LIAFSGDA-PQGTTPRYLLQATQAFYQQEPARSDPMYQKYVQFLSGARPYPN-QGFPQNR

KT--LQSALMDALQ----------------------------------------------

----------

>Sce So ce 56 gi|162453656|ref|YP_001616023.1| thiamine pyridinylase d-proteobacteria [Sorangium cellulosum 'So ce 56']

------------------------------------MRTSVTPERAHSRTREALPAPPSM

DN-MTTI-RRPSACLWTAVLALSAAGCGGVGSTASAPCPPPTPLQAASSIDSAGKPGRTK

LSVALVPWIPDAAGDDFASLRTMIEDGFESQHAAIDLDL----------RLVKSDDSYRN

PARLAGWLAS--G--RHDLVEIDTVVLGDLIDADVLEPWP---SVPER-DYLAAARSAST

EIDRSGKSVWWGVPHLSCGFFLVTRSE-------KLDAAS-SIAGLVK--AARESGKP--

------LLGNFAS--SWDLPALYLNARVDNGLDATSPLALSNAVKPPLHAGSTAALKQLA

ELC--RQ-GDR--NPCIDG---TYDETLE-GPVEEFVKGSAIGYWGYSERLHRTVTLMKA

QGLPVEGLRVSTIPLGTSATPLLFTDAFVRRKG--CSGDPACNAAAAAWAEFITRDDVMR

DLLISRDA-GPTAVPRYLLPATSSAFQAEPIARDALYAALRPLAQSGRSFPS-HASMYER

RS-ALAHLLETQLAAR--------------------------------------------

----------

>Sod 4Rx13 gi|270261863|ref|ZP_06190135.1| hypothetical protein SOD_b00700 enterobacteria [Serratia odorifera 4Rx13]

MSLIYRALFYAFAVILSFFSSTAVVAGQLILKNISLSPITCTVDGWTVSSGSSFDWFIKV

QPGQSFFVGQNTSRPKDPVINWAK--CNNLHTRAMTI--TPSGPNQTLVLNG---QQTQV

LNVSLYPFLPTLPTDNFENLVAYIVQTYQSQHPQVLLNA---------VLNPQVNIYS--

FTELPVLLGND-G---FDVIELDVLYLGFLADNHLINPA----QITGE-APWAVALAGA-

---TYQGQLW-AIPSWLCMDFFYAFDQ-------AIQQQK-TLSKMLNYLNTRTSSVAK-

------MAGSFNG--SWAIPSDYINAYVQTYGYGALQQAMQMP-PDNGVVHQ---LVSLS

DTC---SFNGV--NKCTNN---TYHNAPNGGAEQVFASGQASTDMGFSEQSF-YVNLYSS

AQKPLY---VVPTPWGEHPQPLLFEDAFVSSAAT-CKPGSQCAANAQAFTTLMTGTAMKN

YIVQSKDL-PAGTPWRTLLVATQQFWQQSIIANNSYYQQLSPIFMTAKAFPN-NFTPQSQ

VA--MANGICNALKQQQPNFVCKSNNSTSLLAPSHGSSIINPRSTDSRRGL---------

----------

>Dde G20 gi|78355684|ref|YP_387133.1| ABC-type sugar transport system periplasmic component-like d-proteobacteria [Desulfovibrio desulfuricans subsp. desulfuricans str. G20]

------------------------------------------------------------

--MAYTAIRRRVSVALCMVSACIVSLCMLTGAAGQAFAGSAAAQAFN--------DGRTV

LRVALYPDIPGD----LESMLRWVERHFEAENPDVDLEL---------VAVPVMDMYE--

VSNIASWLTQPVSGGGMHLLEIDSLLLGAAVATGSVAQQ----TLVMP-DWHPAAYATA-

---HVDGRQY-GVTHWLCGYFLMTPHR-------AAAEAD-SMQQMLEALQIVRPEPPY-

------LGADYTS--SWFISGYYLQSWMDNFGRDSVRVGVYAP-VNEVPASA---VGDVA

HMC--VS-RGN--NPCVDG---TYA-DFS-VMVADALQGRLAGLAGFSETMR-EVVAQG-

-GDAAD-WYVTPFVLGPEKDMMLMSDVFVGRKN--MTP--DETDAAERFMRFMLEDSTYA

GILF-----PQGAPPRYVIPARMDVLQEGPFAADVYYSRLRDAIRTAGHFPN-QGVPENR

ER--IFSGVLPYLRDDALPEDFPAKDVKRTSVPVHRKARWQRHKGHFVVDEPMLPLHP--

----------
